# Effect of Duration of Olive Storage on Chemical and Sensory Quality of Extra Virgin Olive Oils

**DOI:** 10.3390/foods10102296

**Published:** 2021-09-28

**Authors:** Annalisa Rotondi, Lucia Morrone, Gianpaolo Bertazza, Luisa Neri

**Affiliations:** Institute for the Bioeconomy, Italian National Research Council, via P. Gobetti 101, 40129 Bologna, Italy; annalisa.rotondi@ibe.cnr.it (A.R.); gianpaolo.bertazza@ibe.cnr.it (G.B.); luisa.neri@ibe.cnr.it (L.N.)

**Keywords:** olive oil, olive storage duration, oil chemical composition, sensory properties

## Abstract

This work considered the influence of the duration of olive storage on the chemical and sensory properties of extra virgin olive oil. In total, 228 batches of olives collected during three successive crop seasons were sampled in seven industrial mills; information about olive batches (variety, harvest date) was collected, together with the produced oils. Four classes of storage times were considered: ≤24 h, 2–3 days, 4–6 days, ≥7 days. The oils’ quality parameters free acidity, peroxide number and K232 increased significantly as storage duration increased, while phenolic content decreased significantly, with a resulting effect on oil stability. The fatty acid composition was not affected by the olive storage period, while α-tocopherol, lutein and β-carotene content decreased as storage duration lengthened. Finally, the main positive sensory attributes (olive fruity, green notes, bitter and pungency) underwent a statistically significant reduction with the increase in storage duration, while the intensity of defects increased, suggesting that the duration of olive storage has an important effect on the quality of the final oil.

## 1. Introduction

Olive oil plays an important role in the diet in Mediterranean countries [1]. Extra virgin olive oil (EVOO) is the only vegetable oil that must be extracted only by mechanical means without any adjuvants [2]. EVOO is therefore, in effect, a fruit juice, hence the phytosanitary state of drupes is the main factor determining the quality of the extracted olive oil [3]. To best preserve the raw material before processing, post-harvest management is strategic to obtain extra virgin olive oils, since during this period oxidation of fat matrix and fermentation can occur [4].

However, in olive-producing regions such as Italy, Spain and Greece, because of the difficulty in synchronizing fruit harvesting and extraction of its oil, the olive sector is often forced to store the fruits piled up, in poor conditions and for periods of up to several weeks. During this period, the fruits suffer mechanical, physicochemical and physiological alterations that may eventually cause the breakdown of their cell structures [5,6]. During prolonged olive storage, anaerobiosis processes can occur in the lower portion of the olives kept inside the containers, and heat production from the respiratory activity may also accelerate fruit deterioration and eventually cause the breakdown of the cell structure [6]. Olive oils obtained from damaged olives present a characteristic high acidity, low oxidative stability and high level of oxidation, due to the increased peroxide value, and specific extinction coefficients at 232 and 270; they can also develop a high content of volatile acids (acetic or butyric) that cause a typical musty smell [7]. These processes will deteriorate the chemical and sensory quality of the resulting EVOO, so in order to better manage the postharvest period, several technological solutions have been proposed such as cold storage of olives [7], storage in a modified atmosphere [8], and other preservation conditions such as storage in sea water, brine or drinking water have also been investigated [9].

The importance of processing olives a short time after harvesting is also linked to the fact that most fruit is harvested mechanically and could, therefore, be internally damaged, more so than in the case of manual harvesting; however, allowing for proper storage conditions the fruits can be stored for several days maintaining the appropriate chemical and sensory quality standards of the final oil. Yousfi et al. [10], in fact, studied the quality of EVOO from mechanically harvested Arbequina olives under different storage conditions, and found that storage at 3 °C for a period of up to 10 days allowed the highest commercial level of oil quality to be maintained.

The problem of synchronization of harvest and transformation phases has not been widely considered in Italy, where this study was carried out in the past due to the production fragmentation, the structure of olive mills (small and widespread) and the presence of different olive cultivars, a factor broadening the collection window. However, the presence of numerous different cultivars on the Italian territory is a characteristic feature of Italian olive growing that increases its sustainability as the loss of biodiversity is an environmental threat. The production of monovarietal olive oils has increased to a great extent lately since the quality of olive oil depends on the olive variety from which it originates [11]. Nowadays, however, the structure of production is changing in Italy, due to the presence of an increasing number of intensive orchards that can exceed the processing capacity of the mills, and therefore synchronization between harvest and transformation should be considered.

The aim of this study was to assess the effect of the duration of olive storage on the chemical and sensory quality of the EVOO, identifying which parameters were most affected by olive storage; in particular, we focused on product parameters that are more easily illustrated to actors in the supply chain (mills, producers and consumers), thus making it easier to understand and assimilate the results.

## 2. Materials and Methods

### 2.1. Olive Fruit Analysis and Oil Sampling

Olive fruits and the corresponding oil samples (*n* = 228) were collected during 3 crop seasons, from seven industrial oil mills located in the Emilia-Romagna region in northern Italy, all equipped with hammer crusher, two-phase decanter, and centrifugation and filtration facilities. Data characterising olive oil samples, such as olive cultivar, harvesting method, and olive storage duration, were collected by interviewing olive growers. Only samples of healthy olives without signs of infection were considered after visual inspection.

Oil samples were poured into dark glass bottles, keeping headspace to a minimum, and stored in the dark in a temperature-controlled cupboard set at 15 ± 1 °C, until chemical and sensory analyses were carried out.

### 2.2. Chemical Analysis of Olive Oils

Free acidity, peroxide value, and UV-spectrophotometric indices (K232, K270) were evaluated in triplicate in line with official methods described in Regulation EC 2568/91 and subsequent amendments [12].

Analysis of fatty acids was carried out according to Regulation EC 2568/91 and subsequent amendments [12] using a Chrompack CP 9000 gas chromatograph with a flame ionization detector (FID), equipped with a capillary column (Stabilwax, Restek Corporation, Bellefonte, PA, USA) and helium as the carrier gas (flow rate = 1 mL min^−1^; split ratio of 1:20, v:v). Chromatographic parameters were as follows: injection and detection temperature 250 °C; 230 °C; column oven temperature, 240 °C. All parameters were determined in triplicate for each sample.

The phenolic fraction was extracted in triplicate from 30 g of oil using 30 mL of methanol. The combined extract was brought to dryness through a rotary evaporator and then suspended in 2 mL 50% methanolic solution. Total phenol content was determined by the Folin–Ciocalteau spectrophotometric method at 750 nm [13] using a spectrophometer (Jasco V-500, Jasco Corporation, Tokyo, Japan).

Quantitative analysis of tocopherols, lutein and β-carotene was carried through olive oil filtration on PTFE (Polytetrafluoroethylene) membrane filter of 25 mm, 0.2 µm pore size (GyroDisc, Orange Scientific, Waterloo, Belgium) and direct injection of 20 µL in HPLC (high-performance liquid chromatography) [14] equipment (LC-10ADvp, Shimadzu, Kyoto, Japan) with a degasser (Flow 154, Gastorr Flom, Tokyo, Japan), a low-pressure gradient unit (FCV-10ALvp, Shimadzu, Kyoto, Japan) and a column oven (CTO-10ASvp, Shimadzu, Kyoto, Japan). Analytes were separated on a C18 column 150 mm × 4.6 mm (Inertsil ODS-2 5U, Alltech, Deerfield, IL, USA); the flow rate was 1 mL min^−1^, the injection volume was 20 μL and the column temperature was 25 °C. The eluent used was: A methanol: water 80:20 (*v*/*v*) and B methanol: tetrahydrofuran 20:80 (*v*/*v*). Quantification of analytes was carried out using their relative analytical standard’s calibration curves all purchased from Merk (Deisenhofen, Germany). Tocopherol quantification was carried out at 295 nm, β-carotene and lutein at 450 nm using a photodiode array detector (UV6000, ThermoQuest, San Jose, CA, USA).

### 2.3. Oil Stability Determination

For determination of oil stability, an eight-channel Oxidative Stability Instrument (OSI) (Omnion, Decatur, IL, USA) was used; the instrument was set at 110 °C and at 120 mL min^−1^ (airflow) [15]. The OSI index was expressed as time (hours and hundredth of hours) and was reported as “OSI time”.

### 2.4. Sensory Analysis

Sensory analysis was performed by the panel of Agency for Agrofood Sector Services of Marche region (ASSAM), a fully-trained analytical taste panel recognized by the International Olive Oil Council (IOC) of Madrid, Spain, and by the Italian Ministry for Agriculture, Food, and Forestry Policy. The panel was composed of 8 assessors, 50% male and 50% female. The method applied was QDA (Quantitative Descriptive Analysis). A profile sheet IOC method T20 n. 15 modified by IBIMET-CNR and ASSAM was used, allowing to obtain a QDA of the oils’ sensory profile and more complete description of the organoleptic properties of the sampled oils: the sensory assessors evaluated direct or retronasal aromatic olfactory sensations (aroma of olive fruity and green notes), gustatory sensations (olive fruity and bitterness) and tactile/kinesthetic sensation (pungency), organoleptic defects (Supplementary Table S1) as well as overall judgment. The sensory assessors had to rate the intensity of the different descriptors on a continuous 0–10 cm scale. Values of median of sensory data were calculated.

### 2.5. Statistical Analysis

The significance of differences at a 5% level was determined by one-way ANOVA using Tukey’s test with Microsoft^®^ Excel 2007/XLSTAT© (Version 2009.3.02, Addinsoft, Inc., Brooklyn, NY, USA). Sensory data were also processed for Principal Component Analysis (PCA) to explore data distribution patterns and to visualize the “distance” between oils produced following the differing storage times.

## 3. Results and Discussion

After interviewing olive growers, it was recorded that the olive and the correspondent Extra virgin olive oil (EVOO) samples collected (*n* = 228) were mainly composed of mixed varieties (blends) (45%), while the remaining samples were monovarietal from cv. Nostrana di Brisighella (25%), cv. Correggiolo (16%), cv. Leccino (8%) and other minor cultivars (6%). Furthermore, it was assessed that the olives’ storage method was the same for all the analyzed samples: fruits were stored in small plastic bins with holes to allow for ventilation, and never in stacks nor in plastic or jute bags.

Olive storage duration before technological transformation ranged from 0 to over 7 days: chemical and sensory data were thus processed dividing them into four classes of storage times: ≤24 h, 2–3 days, 4–6 days, ≥7 days. Only 39% of olive samples were processed within 24 h, while 23% and 20% of olive samples were stored between 2 and 3 days and between 4 and 6 days, respectively; finally, 18% of samples were processed after 7 days of storage.

The content of free acids is an important quality factor, extensively used as the major criterion for the classification of olive oil at various commercial grades [16]. The values of free acidity, peroxide number, and K232 increased significantly along with the increase in olive storage duration (Table 1). There was a free acidity increase from 0.30% to 0.56% during the olive storage period studied; peroxide number from 6.96 to 9.56 mEqO_2_kg^−1^, K232 from 1.48 to 1.66 while K270 was not affected by time of storage probably because indicates secondary oxidation. It is interesting to note that all these values fall within the legal limit of the classification of extra virgin olive oil. This indicates that, although the oxidation process starts to take place during the olives’ storage time, the phytosanitary state and the integrity of the raw material affect the speed of this process. The total phenol content of oil samples suffered a progressive reduction as olive storage duration proceeded: as observed in Table 1, oils produced within 24 h from the olive collection had a phenolic content of 243 mg kg^−1^ of gallic acid while oils produced from olives stored for over 7 days presented 143.6 mg kg^−1^ of gallic acid, a decrease of 41%. This phenol loss could be attributed both to bacteria and fungi proliferation on cellular fluid exuding from damaged fruits [17] and to endogenous oxidoreductases [18] as suggested by Clodoveo et al. [5], results of which were consistent with the data presented here. This impoverishment in the phenol fraction also affected the oils’ stability, with a reduction from 28 h in oils produced within a day to 19 h in oils obtained from olives stored for more than a week, with a decrease of 30% (Table 1). These results agree with the studies of Vichi et al. [19] and Youssef et al. [20]. Our results partially agree with Pereira et al. [21], which found a significant decrease in oil stability during the first period of storage (0–7 days), while for peroxide number, free acidity and K232 and K270 the values were not significantly affected by storage duration. As explained by Pereira himself [21], the verified decrease in stability was due to the consumption of minor compounds such as phenols and tocopherols, that hindered the formation of peroxides.

Fatty acid and sterolic profile can be used as an exceptional compositional marker for olive oil authenticity [11]. Fatty acid composition of all of the EVOO samples extracted from olives after different storage duration was characterized by the high oleic acid content (Table 2), coherent with the cold climate of the region; the relationship between fatty acid composition and climate is well known [22]. Several studies reported that fatty acid composition in oils did not show any change as the period for which olives were stored prior to crushing increased [10,23] and neither did they even when the olive storage period was very long, e.g., 45 days, as reported by Gutierrez et al. [24]. However, other studies [20,21] found differences in fatty acids content during storage, in agreement with our results. Specifically, we found differences in the content of C16:0, C18:1 and C18:2. In fact, the C16:0 content tended to increase as the olive storage period lengthened, while concentrations of C18:1 and C18:2 did not show a clear trend.

The content of α-tocopherol, the naturally occurring form of vitamin E assimilated by the human body, found in oils obtained from olives belonging to different conservation classes showed a statistically significant decreasing trend (Table 3). Vitamin E is an antioxidant, working as peroxyl radical scavenger that terminates chain reactions [25]. As it is well documented, oxidation phenomena are the main cause of tocopherol degradation [26]; the data here presented showed that milling olives within 24 h from the collection was the only way to protect the tocopherol fraction. In this study, the decrement in α-tocopherol content found after 7 days of olive storage was about 17%, consistent with the reduction of 22% of total tocopherol content found by Yousfi et al. [10] for cv. Arbequina and Pereira and colleagues [21] for cv. Verdeal Transmontana. An important and significant decrease in α-tocopherol after a short (48 h) olive storage period was as well found for cv. Nostrana di Brisighella oils while not for cv. Correggiolo oils [27].

In the present study, carotenoid pigments decreased during the first three storage times analyzed, in agreement with other works [28,29]. At the last time of storage duration analyzed (>7 days), olive oils showed an increase in carotenoid content, in line with Yousfi and colleagues [10], who hypothesized a greater extractability of the pigments in olives during storage, due to the degradation of the chloroplast membranes; the degradation was found to be a consequence of the growing dehydration of the olives during storage [10].

A correlation analysis was carried out to quantify the intensity of the connection between EVOO properties and olive storage duration (Table 4). A positive correlation (Pearson) with storage duration was found for the parameter acidity, peroxide number, K232 and palmitic acid content, while palmitoleic and stearic acid, total phenol content and oil stability exhibited a negative correlation, diminishing with the increase in storage duration. In the case of fruits left for long periods before transformation, in the produced oils was observed, in addition to an increase in free acidity, even a gradual depletion in the content of oleic acid and total phenols, with the consequent reduction in stability during storage. It is also important to underline the correlation (r = 0.388, *p* ≤ 0.0001) between oleic acid and OSI found in this work (data not shown). In fact, a high concentration of oleic acid enhances the oil stability and EVOO oxidative stability is mainly linked to its fatty acid composition, therefore the induction period is the result of the fatty acid composition and the simultaneous activity of various prooxidant and antioxidant factors endogenous in the oils [30].

The sensory profile was modified according to the olive storage duration before processing, with the main positive attributes (olive fruity, green notes, bitterness and pungency) undergoing a statistically significant reduction with the increasing of olive storage days (Table 5). This result was in line with the previously recorded decrease in total phenols: the typical bitter taste and pungent note of fresh EVOO rich in total phenols decreased in intensity as olive storage duration lengthened. The intensity of the defects perceived by the sensory assessors increased with the progress of the days of olives storage (Table 5). Even in oils processed within 24 h, an intensity of the defect of 0.29 was recorded, probably attributable to the percentage of olives harvested late and thus overripe. In detail, the perceived defects were attributable to incorrect management of the raw material: in fact, they were 67% for the fusty defect, 29% for the vinegary and only 4% for the musty defect. During prolonged olive storage duration, the drupe tissues are damaged, resulting in the secretion of fluids favoring the growth of undesirable microorganisms [17]; increased temperature can also increase drupe respiratory activity, leading to undesirable metabolic processes accelerating fruit deterioration and characterized by the fusty sensory defect [31].

**Figure 1:**
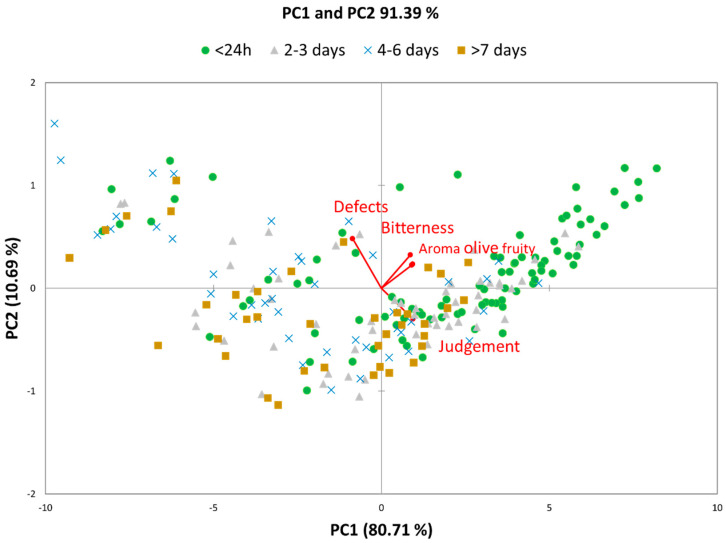
Principal component analysis (PCA) plot of sensory data.

The Principal Component Analysis (PCA) of the sensory data explained 91.4% of the variability and confirmed the strong influence of olive storage duration on the sensory characteristics of the oils produced (Figure 1). Most of the samples that transformed within 24 h are, in fact, positioned in the first quadrant of the PCA, showing the greatest intensities of positive attributes such as the aroma of olive fruity and green notes and bitterness. The small percentage of oil samples that, despite having been transformed within 24 h, are positioned in the fourth quadrant relative to the presence of sensory defects, is probably attributable to oils produced from overripe olives [32].

## 4. Conclusions

The objective of this study, unlike other studies which investigated preprocessing storage as a way to modulate a positive reduction in the bitter taste of phenol-rich varieties with the aim of improving consumer acceptance [31], was to identify the criticality of the olive storage phase, highlighting its influence on the depletion of the EVOOs’ chemical and sensory characteristics.

The authors are aware that this experimental design included many variables that affect the final quality of the oil: cultivar, ripeness, cultivation environment, seasonality and variation of technological parameters of extraction. However, thanks to the high rate of sampling, repeated for three consecutive production years, the single effect of the different variables was reduced. From this study, it emerged that some quality, nutritional and sensory parameters were affected by olive storage duration, independent of the varietal composition of the starting material. However, variety and ripeness degree influence the time window available to leave the olives on trees [33]. The knowledge of the effect of the harvest time window (early harvest or late harvest) on the olive oil final quality is important especially in years with late fly attacks, when it is recommended to harvest early rather than treating with chemicals, since a sustainable olive growing satisfies consumers who are increasingly attentive to the consumption of genuine and healthy products.

This study was carried out on purpose in a practical context characterized by all the limits listed above, thus the results provide an important photograph of the critical points of the olive storage phase from harvesting to pressing. By acting on the critical points, it is possible to improve the chain of olive oil production.

Free acidity, peroxide number, K232, total phenols, stability, α-tocopherol, lutein, β-carotene and organoleptic properties significantly decreased between the first and second storage interval, thus after 24 h of olive storage the final EVOO’s quality was already substantially impoverished. Many specifications for PDO and PGI productions indicate 48 h as the maximum allowed storage duration; however, it is important to underline that by keeping storage within 24 h it is possible to maximize the potential of the olives, thus producing the oils with the highest nutritional and sensory properties expected by selecting cultivars known for the high quality of the final product. While PDO and IGP oils are produced according to strict production regulations, this study is aimed at blends productions that represent most of the processed olives in all of the Italian regions: for this kind of production the improving of the crucial phase of olive storage duration is important, and the results of this study are clearly significant for their applicability. The purpose of this work was to provide guidelines for obtaining a high-quality product at the time of processing, initial high quality being pivotal during the oil storage phase. The associations of olive producers guarantee their associates the supply of plastic aerated bins, together with guidelines aimed at reducing the olives’ storage times from harvesting to processing. Ensuring the chemical and sensory oil quality during shelf life is, in fact, becoming the purpose of the most recent labeling regulations [34]: the community regulation states that what is indicated on the label should correspond with what is expected at the end of the product’s shelf life.

## Figures and Tables

**Table 1:** Quality index of oil samples extracted after different olive storage duration. Values are mean ± standard deviation. Values followed by different letters in the same column (a, b, c) were significantly different according to Tukey’s test (*p* < 0.05).

Storage Classes	Free Acidity	Peroxide Number	K232	K270	Total Phenol	OSI
<24 h	0.30 ± 0.09 b	6.96 ± 2.17 b	1.48 ± 0.18 b	0.08 ± 0.02	246.00 ± 104.02 a	28.28 ± 10.83 a
2–3 days	0.37 ± 0.17 b	9.03 ± 2.76 a	1.64 ± 0.23 a	0.09 ± 0.02	202.51 ± 90.14 b	22.60 ± 7.53 b
4–6 days	0.51 ± 0.36 a	8.67 ± 2.94 a	1.55 ± 0.25 ab	0.08 ± 0.02	179.22 ± 83.49 bc	16.90 ± 5.74 b
>7 days	0.56 ± 0.43 a	9.56 ± 2.64 a	1.66 ± 0.29 a	0.09 ± 0.02	143.60 ± 63.39 c	19.91 ± 10.08 b
*p*-value	<0.0001	<0.0001	<0.0001	0.394	<0.0001	<0.0001

Free acidity is expressed as g/100 g of oleic acid; peroxide number as mEq O_2_kg^−1^ oil; OSI, oxidative stability index, as hours; total phenol as mg kg^−1^ of gallic acid.

**Table 2:** Fatty acid composition of oils produced by olives after different olive storage duration. Values are mean ± standard deviation. Values followed by different letters in the same column (a, b) were significantly different according to Tukey’s test (*p* < 0.05).

Storage Classes	C16	C16:1	C18	C18:1	C18:2	C18:3
<24 h	12.67 ± 0.96 b	1.25 ± 0.26	2.09 ± 0.23	75.39 ± 1.48 a	6.74 ± 0.98 b	0.74 ± 0.09
2–3 days	13.07 ± 0.73 ab	1.2 ± 0.18	2.03 ± 0.25	75.02 ± 1.53 ab	6.97 ± 1.07 ab	0.71 ± 0.07
4–6 days	13.17 ± 1.05 a	1.2 ± 0.17	2.04 ± 0.19	74.49 ± 1.80 b	7.31 ± 1.18 a	0.73 ± 0.08
>7 days	13.15 ± 0.76 a	1.15 ± 0.16	1.99 ± 0.16	75.17 ± 1.61 ab	6.75 ± 1.16 ab	0.73 ± 0.08
*p*-value	0.004	0.071	0.089	0.020	0.026	0.213

**Table 3:** Tocopherol and carotenoid content in oil samples after different olive storage durations. Values are mean ± standard deviation. Compounds are expressed as mg of relative standard compound per kg of oil. Values followed by different letters in the same column (a, b) were significantly different according to Tukey’s test (*p* < 0.05).

Storage Classes	α-Tocopherol	β+γ-Tocopherol	Lutein	β Carotene
<24 h	184.25 ± 36.59 a	8.29 ± 1.84	1.67 ± 0.62 a	1.05 ± 0.63 a
2–3 days	165.63 ± 32.8 b	8.31 ± 2.32	1.34 ± 0.94 b	0.80 ± 0.68 b
4–6 days	160.98 ± 32.85 b	8.33 ± 2.07	1.19 ± 0.42 b	0.64 ± 0.41 b
>7 days	157.77 ± 30.96 b	8.24 ± 1.80	1.46 ± 0.46 ab	0.82 ± 0.34 b
*p*-value	<0.0001	0.988	0.000	0.000

**Table 4:** Pearson correlation between oil chemical parameters and olive storage duration.

Variable	r	*p*
Acidity	0.244	0.000
Peroxide number	0.312	<0.0001
Total phenols	−0.319	<0.0001
C 16	0.149	0.031
C 16:1	−0.184	0.007
C 18	−0.139	0.044
C 18:1	−0.009	0.894
C 18:2	−0.038	0.587
C 18:3	−0.021	0.757
OSI	−0.262	0.000
K_232_	0.295	<0.0001
K_270_	0.035	0.615

**Table 5:** The intensity of sensory attributes (aroma of olive fruity and green notes, flavor of bitterness, pungency and defect) of oil samples related to different olive time storage. Values are median ± standard deviation. Values followed by different letters in the same column (a, b, c) were significantly different according to Tukey’s test (*p* < 0.05).

Storage Classes	Olive Fruity	Green Notes	Bitterness	Pungency	Defect
<24 h	2.25 ± 0.5 a	1.36 ± 0.65 a	2.16 ± 0.76 a	1.93 ± 0.52 a	0.29 ± 0.55 b
2–3 days	1.98 ± 0.45 b	0.92 ± 0.54 b	1.76 ± 0.62 b	1.74 ± 0.46 ab	0.34 ± 0.50 b
4–6 days	1.79 ± 0.41 b	0.64 ± 0.51 b	1.35 ± 0.53 c	1.43 ± 0.46 c	0.79 ± 0.76 a
>7 days	1.75 ± 0.38 b	0.57 ± 0.43 b	1.4 ± 0.65 bc	1.41 ± 0.51 bc	0.57 ± 0.64 b
*p*-value	<0.0001	<0.0001	<0.0001	<0.0001	<0.0001

## Supplementary Materials

The following are available online at https://www.mdpi.com/article/10.3390/foods10102296/s1. Table S1: Definition of sensory descriptors.
