# Supplementary material for: Effect of Duration of Olive Storage on Chemical and Sensory Quality of Extra Virgin Olive Oils
Source: Foods. 2021 Sep 28;10(10):2296. doi: 10.3390/foods10102296 (PMC8534741; doi:10.3390/foods10102296)
Supplement: Supplementary file 1 [file foods-10-02296-s001.zip › foods-1404354-supplementary.pdf]

Table S1. Definition of sensory descriptors.

Descriptors	Definition	Reference
Olive fruity	range of smells (dependent on variety) characteristic of oil from healthy fresh fruit, green or ripe, perceived directly and/or retronasally	COMMISSION REGULATION (EC) No 640/2008
Bitter	characteristic primary taste of oil from green olives or olives turning colour. It is detected by the circumvallate papillae in the "V"region of the tongue.	COMMISSION REGULATION (EC) No 640/2008
Pungent	tingling sensation characteristic of oil made at the beginning of the season mainly from olives that are still green. It can be perceived throughout the mouth cavity, particularly in the throat.	COMMISSION REGULATION (EC) No 640/2008
Green notes	Complex olfactory sensation reminiscent of the typical odour of fruit before it ripens	COI/T.20/Doc. no. 22
Overall judgment	Score that expresses the quality level of the oil on a scale from 0 to 10 where 6.5 is the limit between the presence and absence of sensory defect	
Fusty	characteristic flavour of oil from olives that have been piled or stored in such a way as to have reached an advanced stage of anaerobic fermentation, or of oil which has been left in contact with the sediment that settles in underground tanks and vats and which has also undergone a process of anaerobic fermentation.	COMMISSION REGULATION (EC) No 640/2008
Musty-humid-earthy	characteristic flavour of oil from olives in which large numbers of fungi and yeasts have developed as a result of storage for several days in humid conditions.	COMMISSION REGULATION (EC) No 640/2008
Winey-vinegary	characteristic flavour of certain oils reminiscent of wine or vinegar. This flavour is mainly due to the aerobic fermentation of the olives or of olive paste left on pressing mats which have not been properly cleaned, leading to the formation of acetic acid, ethyl acetate and ethanol.	COMMISSION REGULATION (EC) No 640/2008

Rancid	favour of oil that has undergone an intense process of oxidation.	COMMISSION REGULATION (EC) No 640/2008
